# Sequence and cultivation study of *Muribaculaceae* reveals novel species, host preference, and functional potential of this yet undescribed family

**DOI:** 10.1186/s40168-019-0637-2

**Published:** 2019-02-19

**Authors:** Ilias Lagkouvardos, Till R. Lesker, Thomas C. A. Hitch, Eric J. C. Gálvez, Nathiana Smit, Klaus Neuhaus, Jun Wang, John F. Baines, Birte Abt, Bärbel Stecher, Jörg Overmann, Till Strowig, Thomas Clavel

**Affiliations:** 1 ZIEL - Institute for Food & Health, Technical University of Munich, Freising, Germany; 2 Department of Microbial Immune Regulation, Helmholtz Centre for Infection Research, Braunschweig, Germany; 3 Functional Microbiome Research Group, Institute of Medical Microbiology, RWTH University Hospital, Pauwelsstrasse 30, 52074 Aachen, Germany; 4 Max Planck Institute for Evolutionary Biology, Plön, Germany; 5 Institute for Experimental Medicine, Kiel University, Kiel, Germany; 6 Leibniz-Institute DSMZ - German Collection of Microorganisms and Cell Cultures, Braunschweig, Germany; 7 Max von Pettenkofer Institute of Hygiene and Medical Microbiology, Faculty of Medicine, LMU Munich, Munich, Germany; 8 German Center for Infection Research (DZIF), partner sites Hannover-Braunschweig and Munich, Germany

**Keywords:** Mouse gut microbiota, Bacterial diversity, *Bacteroidetes*, Family S24-7, Homeothermaceae, *Muribaculaceae*, Metagenomic species, Cultivation

## Abstract

**Background:**

Bacteria within family S24-7 (phylum *Bacteroidetes*) are dominant in the mouse gut microbiota and detected in the intestine of other animals. Because they had not been cultured until recently and the family classification is still ambiguous, interaction with their host was difficult to study and confusion still exists regarding sequence data annotation.

**Methods:**

We investigated family S24-7 by combining data from large-scale 16S rRNA gene analysis and from functional and taxonomic studies of metagenomic and cultured species.

**Results:**

A total of 685 species was inferred by full-length 16S rRNA gene sequence clustering. While many species could not be assigned ecological habitats (93,045 samples analyzed), the mouse was the most commonly identified host (average of 20% relative abundance and nine co-occurring species). Shotgun metagenomics allowed reconstruction of 59 molecular species, of which 34 were representative of the 16S rRNA gene-derived species clusters. In addition, cultivation efforts allowed isolating five strains representing three species, including two novel taxa. Genome analysis revealed that S24-7 spp. are functionally distinct from neighboring families and versatile with respect to complex carbohydrate degradation.

**Conclusions:**

We provide novel data on the diversity, ecology, and description of bacterial family S24-7, for which the name *Muribaculaceae* is proposed.

**Electronic supplementary material:**

The online version of this article (10.1186/s40168-019-0637-2) contains supplementary material, which is available to authorized users.

## Introduction

Bacterial diversity on earth is tremendous and only a small fraction has been described so far [19, 63]. Hence, it is very important to study this wealth of diversity to be capable of dissecting fundamentally important processes such as nutrient cycles [14] and the health-modulating functions of host-associated microbial communities [26]. The mammalian gut is colonized by hundreds of different bacterial species, the majority of which belongs to the phylum *Firmicutes* and *Bacteroidetes* [12, 48]. *Bacteroidales* is one of the most prevalent orders among intestinal *Bacteroidetes*, including dominant and functionally important members of gut microbiomes such as species of the family *Bacteroidaceae*, *Barnesiellaceae*, *Porphyromonadaceae* (e.g., *Parabacteroides* spp.), *Prevotellaceae*, and *Rikenellaceae* (e.g., *Alistipes* spp.). Sequence-based surveys of the mouse gut microbiota have consistently revealed the existence of another dominant family of gut *Bacteroidales*, so far designated as family S24-7. The first trackable report of this family by Salzman and colleagues, who referred to it as MIB (mouse intestinal bacteria), mentioned a relative abundance of 20 to 30% total bacteria in the mouse gut as detected by fluorescence in situ hybridization [55]. In 2014, data by Seedorf and colleagues [56] supported the concept that S24-7 members are well adapted for colonization of the mouse intestine by showing that several molecular species were capable of outcompeting colonization of germfree mice by human gut bacteria after a period of 14 days. Recently, Ormerod and colleagues [44] performed a study based on 30 genomes of S24-7 members assembled from shotgun sequence datasets. Thereby, the authors reported their occurrence in homeothermic animals and demonstrated the presence of a substantial and versatile set of carbohydrate-active enzymes in the genomes analyzed. Concurrently, we published the first cultured member of the family, *Muribaculum intestinale* DSM 28989^T^, as part of the mouse intestinal bacterial collection (www.dsmz.de/miBC) [31], and included the species in the Oligo-MM, a minimal bacterial consortium used for standardized colonization of germfree mice [11].

In spite of these recent findings, family S24-7 still has no valid name with standing in nomenclature. Confusion in the literature arises from various classifications in databases: e.g., *Porphyromonadaceae* in RDP [68] and S24-7 group in SILVA [50], with the addition of the non-validated name “Homeothermaceae” as proposed by Ormerod and colleagues [44]. Moreover, our understanding of its ecology and diversity is poor. These limitations prevent proper interpretation of an ever-increasing number of sequencing data and hamper full appreciation of the role of these important members of the gut microbiota in animals. For these reasons, we undertook a comprehensive investigation of the bacterial family at multiple levels: large-scale 16S rRNA gene survey, genomic and metagenomic studies, and culture-based analysis. Thereby, we provide a detailed overview of S24-7 diversity, novel insights into its ecology and functional potential, a taxonomic description of two novel genera, and propose the name *Muribaculaceae* to accommodate members of the family.

## Materials and methods

### Bacterial isolation and identification

All strains were grown under strict anaerobic conditions, checked for purity by re-streaks and microscopic observation prior to identification by 16S rRNA gene sequencing. A 16S rRNA gene sequence identity of ≤ 94.5% was considered strong evidence for different genera [69]. Potential novel taxa were deposited at the Leibnitz Institute DSMZ-German Collection of Microorganisms and Cell Cultures for long-term storage and for determination of cellular fatty acid compositions as described previously [31]. Other criteria used for taxonomic description of isolates are detailed below in the section “Genome-based taxonomy”. Strains B1404 and B1117^T^ were isolated from feces of wildtype C57BL/6 mice housed at the Leibniz Institute in Borstel, Germany, using Columbia blood agar. Strains YL5 and YL7 were isolated in the same manner as the type species *M. intestinale* DSM 28989^T^ [31]. For strain 129-NLRP6, the caecal and colonic content of *Nlrp6*^*−/−*^ C57BL6/N mice housed at the Helmholtz Centre for Infection Research (Braunschweig, Germany) [51] was diluted 1:25 (*w*/*v*) into thioglycollate broth (BD Bioscience) under anaerobic conditions and filtered through a 70-μm cell strainer. A dilution-to-extinction approach allowed identifying 96-well plates with a maximum of 30% wells showing detectable growth after 1 day. Cell suspensions from these wells were streaked onto agar to isolate single colonies. Bacteria were then grown in/on mucus medium, consisting of 18.5 g/L BHI (Sigma-Aldrich), 15 g/L Trypticase Soy Broth (Oxoid), 5 g/L yeast extract (Roth), 0.025% mucin (Sigma-Aldrich), 2.5 g/L K_2_HPO_4_ (Roth), 1 g/L glucose (Roth), 1 mg/L hemin (Sigma-Aldrich), 0.4 g/L Na_2_CO_3_ (Merck), 0.5 g/L L-cysteine HCl (Sigma-Aldrich), 0.5 g/L menadione (Sigma-Aldrich), and 3% fecal calf serum.

### 16S rRNA gene diversity of the family

All non-chimeric 16S rRNA gene sequences classified as S24-7 in SILVA were collected (*n* = 29,743), combined with those of the first cultured member of the family *M. intestinale* [31] as well as isolates and metagenomic species from the present study (*n* = 26 and 105, respectively), and clustered at family level (ca. 90% similarity) using CROP [18]. This approach resulted in the creation of multiple family-level clusters, only one of which contained the sequences of the type species *M. intestinale*, the new isolates (described below), and all metagenome-derived S24-7 species. The other clusters probably represent other S24-7-like families that were not considered further. Only those sequences belonging to the congruent S24-7 family cluster were kept (*n* = 9397), filtered for size (> 1200 nt), and ambiguous sites (only A, G, C, and T allowed), resulting in 7784 remaining sequences that were clustered at the species level (ca. 97% similarity). The centroids of resulting molecular species clusters were first aligned using SINA [47] followed by manual refinement. The final alignment was used to construct a phylogenetic tree based on the neighbor-joining algorithm in MEGA7 [28]. The topology of the tree was annotated using iToL [34]. A subtree was constructed with sequences from clusters containing members with available genomic information using the UPGMA method also in MEGA7.

### IMNGS-based 16S rRNA amplicon survey

In order to analyze the ecological distribution and abundance of S24-7 members in various sample types, we used amplicon data from the 93,045 samples contained in IMNGS, build 1706 [30]. For every operational taxonomic unit (OTU) sequence in every sample, a similarity search was performed against a local, comprehensive 16S rRNA gene database containing the S24-7 molecular species centroids (described in the previous section) and a total 14,734 16S rRNA gene sequences from cultured species [32] using BLAST [3]. Amplicons with > 97% sequence similarity over > 90% of their sequence length to any S24-7 species centroid were collected (*n* = 545,544) and classified using a local SINA server. All amplicons confirmed to be S24-7 members (*n* = 337,137) were considered as evidences for the presence of the corresponding closest centroids in the particular sample of origin of each amplicon. This allowed estimation of the detailed prevalence and abundance of the predicted S24-7 species in all available sample types.

### Shotgun sequencing of mouse gut samples

Feces and gut contents from three distinct locations (ileum, caecum, and colon) were collected from mouse lines either housed at the Helmholtz Centre for Infection Research (HZI, Braunschweig, Germany) or obtained from different providers (Janvier, Harlan, National Cancer Institute). DNA was isolated using an established protocol [9]. Briefly, 500 μL extraction buffer (200 mM Tris, 20 mM EDTA, 200 mM NaCl, pH 8.0), 200 μL 20% SDS, 500 μL phenol:chloroform:isoamyl alcohol (24:24:1), and 100 μL zirconia/silica beads (0.1 mm diameter) were added and samples were homogenized using a BioSpec bead-beater for 2 min. DNA was precipitated using absolute isopropanol, washed with 70% (*v*/*v*) ethanol, and re-suspended in TE-buffer with 100 μg/mL RNase I prior to purification on columns. Metagenomic DNA was quantified and diluted to 25 ng/μL. For library preparation, metagenomic DNA (60 μL) was sheared by sonication (Covaris) with the following specifications: processing time, 150 s; fragment size, 200 bp; intensity, 5; duty cycle, 10. DNA fragments were selected by size using AMPure XP beads (55 and 25 μL for first and second selection, respectively) and 500 ng purified DNA was used for library construction using the NEBNext Ultra DNA Library Prep Kit according to the manufacturer’s instructions (New England Biolabs). Adaptor enrichment was performed by means of seven cycles of PCR using the NEBNext Multiplex Oligos for Illumina (set 1 and 2) (New England Biolabs). Libraries were sequenced using the Illumina HiSeq system (PE100) according to the manufacturer’s instructions.

### Metagenomic species (MGS) reconstruction

Metagenomic reads from all libraries (*n* = 298) were processed in a single all-in-one assembly approach using Megahit [35]. After size filtering (contigs > 1000 nt were kept), all reads were mapped to the contigs using BWA [36] and results were transformed and indexed to bam-format using Sambamba [65]. Sequences were binned using MetaBAT (version 0.32) [25] with the following parameters: -verysensitive -pB 20 -B 100 -minclustersize 200,000. Resulting clusters were quality-controlled using CheckM [45]: bins with marker gene completeness minus contamination ≥ 80% were considered as high-quality metagenome species (MGS). Metagenemark [71] was used for protein prediction, discarding ORFs < 100 bp. Full-length 16S rRNA gene sequences were reconstructed using RAMBL [70] with all metagenomic libraries as input (all-in-one assembly approach) and were then linked to their corresponding MGS by means of an integrated score combining mapping- and correlation-based associations [2, 41]. MGS/16S rRNA gene sequence pairs were manually curated via taxonomy filtering (both had to be assigned to the S24–7 family) and congruence of phylogenetic placement. All the details of this method are available elsewhere [33].

### Genome sequencing and processing

A total of 26 draft genomes were obtained in the present study, including the five isolated strains that were maintained in culture and 21 strains that could be isolated and grown for the generation of biomass but were then lost during sub-culturing. DNA libraries were prepared using either (i) the TruSeq DNA PCR-Free Sample Preparation Kit (Illumina) following a protocol optimized (DNA shearing and fragment size selection) to improve assembly quality [20] when the amount of DNA template was sufficient (≥ 3 μg) or (ii) using the NEB Next Ultra II DNA Library Prep Kit (New England Biolabs, ref. E7645S) for lower amounts. Libraries were sequenced using the Illumina MiSeq system according to the manufacturer’s instructions. Reads were assembled using Spades v3.6.1 [7] with activated BayesHammer tool for error correction and MismatchCorrector module for post-assembly mismatch and indel corrections. Assemblies were predicted using Quast v3.1 [17]. Proteins were annotated using Prodigal [21] and annotated using BLASTP [4] against the KEGG gene database (01/2018) [24] and CAZy database [37] following the best hit approach (*e* value 0.001). KEGG Orthology annotation was used to reconstruct KEGG module completeness [22].

### Genome-based taxonomy

For taxonomic description, ORFs within each of the isolates’ assemblies were identified using Prodigal version 2.6.3 [21] and then annotated using the KEGG web service BlastKOALA [23] against both the eukaryotic family and prokaryotic genus databases. To identify the presence and functional content of plasmids in all 27 isolate-derived S24-7 genomes, Recycler [52] was used after re-assembly with plasmidSPAdes [6] using default settings. Assembled plasmids were then annotated against the KEGG database using BlastKOALA.

For phylogenomic placement, representative species genomes within the order *Bacteroidales* (*n* = 275) were downloaded from the NCBI assembly database. These genomes along with *Fibrobacter succinogenes*, which was used as an out-group allowing the tree to be rooted, and the novel genomes generated in the present study were analyzed with PhyloPhlAn version 0.99 [57]. This allowed for placement of the novel genomes within *Bacteroidales* using the 400 most conserved proteins across genomes. The produced tree was then visualized using iTOL [34] with branch length representing amino acid substitutions per position.

For digital DNA-DNA hybridization (dDDH), the Genome-to-Genome Distance Calculator 2.0 (GGDC), a web service freely available at http://ggdc.dsmz.de, provided a genome sequence-based delineation of (sub-)species by reporting dDDH estimates as well as their confidence intervals [39]. A dDDH value of < 70% indicated affiliation of an isolate to a novel species. The difference in genomic G + C content was also used to delineate species. Because within-species differences in the genome-based G + C content of DNA are almost exclusively < 1% [40], larger differences strongly supported the status of distinct species. Finally, the percentage of conserved proteins (POCP) analysis was done using the IMG software tool Genus definition [38, 49], considering genus delineation at POCP values of approximately 50%.

## Results

### S24-7 diversity and ecology by large-scale 16S rRNA gene sequence analysis

**Fig. 1:**
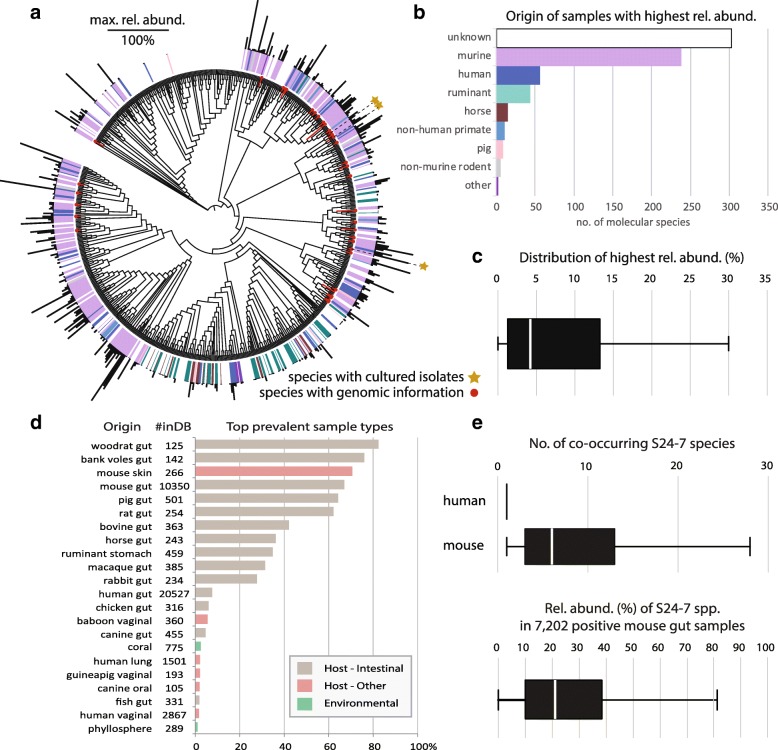
Diversity and ecology of family S24-7 species. In all figure panels, only samples with S24-7 matches ≥ 0.25% relative abundance were considered for plotting, a threshold of confidence below which the risk of including spurious OTUs in the analysis increases substantially. **a** Cladogram of the family’s diversity (based on a Neighbor-Joining tree) as supported by existing full-length 16S rRNA gene sequences (*n* = 7784 after quality checks). The colored ring (color code corresponds to panel **b**) indicates the origin of samples recorded in IMNGS-derived samples (www.imngs.org) with the highest relative abundance for the given molecular species. The outer black bars represent the values of these maximum relative abundances (see scale). Terminal tree branches colored in red indicate species with existing genomic information, corresponding to reconstructed metagenomic species, short-term isolates, or those able to be maintained in culture and taxonomically described in this study (yellow stars). **b** Summary of sample origins recorded in IMNGS with the highest relative abundance of sequences for the 685 molecular species shown in panel **a**. The category “unknown” corresponds to S24-7 species for which the full-length 16S rRNA gene sequence returned no match to any of the amplicon sequences in existing IMNGS samples at > 97% sequence similarity over > 90% sequence length. **c** Distribution of highest relative abundances across all molecular species. Only positive species were considered for plotting, i.e., species of unknown origin (no sequences detected in amplicon datasets) or occurring at < 0.25% relative abundance were ignored. **d** Prevalence of S24-7 spp. in samples of different origins. Each bar represents the percentage of samples in the given category that were positive for S24-7 species. The column labeled “#inDB” provides the total number of samples per category. **e** Number of co-occurring S24-7 species in the tested human and mouse gut samples and distribution of the cumulative relative abundance of the family in the mouse gut (positive samples only)

Full-length (> 1200 nt) 16S rRNA gene clustering of a total of 7784 sequences (see selection criteria in the “Materials and methods” section) generated 685 species-level clusters within family S24-7 (Fig. 1a). IMNGS-based investigation revealed that many of these taxa (*n* = 302) remained orphan in terms of ecological habitats (white in the figure) (Fig. 1a, b). Of note, although these species were each represented by a cloned sequence (either unique or picked as representative of the species-cluster) with a known origin of isolation, we refer to them as being orphans because the source of a single sequence does not necessarily reflect the true ecology of a species as assessed here in a large-scale manner. These orphan species had not a single hit in amplicon datasets (93,045 samples analyzed), which suggests that either they are sub-dominant community members or that the high species diversity within family S24-7 is characterized by local islands of diversity (e.g., mouse facilities) not yet represented in our amplicon database. For other species-level clusters (those with amplicon hits), the majority (34.9%, 239 species) were characterized by highest relative abundance in mice, followed by humans (8.2%, 56 species), ruminants (6.4%, 44 species), and other animals (< 20 species each) (Fig. 1b). Only twelve species were characterized by highest occurrence in samples other than host-associated, albeit mostly manure or wastewater treatment plants and only one species from coral reef. Members of the S24-7 family showed a wide span of highest relative abundances when considering all species, yet three quarters of the values laid between 2 and 13% relative abundance (Fig. 1c).

Looking in detail at the prevalence of S24-7 species in all sample categories available in the IMNGS database showed that highest prevalence (> 50% positive samples) was found in the intestine of rodents (e.g., woodrat, bank voles, mouse, rat), with ca. 67% (6934 of 10,350) mouse gut samples being positive for S24-7 members. In contrast, human gut samples were characterized by a prevalence of only 7% (1556 of 20,527) (Fig. 1d). Another noticeable habitat besides mice was the pig intestine: 66% prevalence, yet in only 501 samples available in the IMNGS database. In the mouse gut, median relative abundance in those samples containing S24-7 spp. was approximately 20% total sequences, and the number of co-occurring species in one given sample was on average 8.7 (median = 6) (Fig. 1e). These data clearly demonstrate the high prevalence and the dominance (whenever present) of family S24-7 members in the mouse intestine and suggests that broad diversity of the family allows functional co-existence of species thanks to different niche occupancies.

### Functional features of the family

**Fig. 2:**
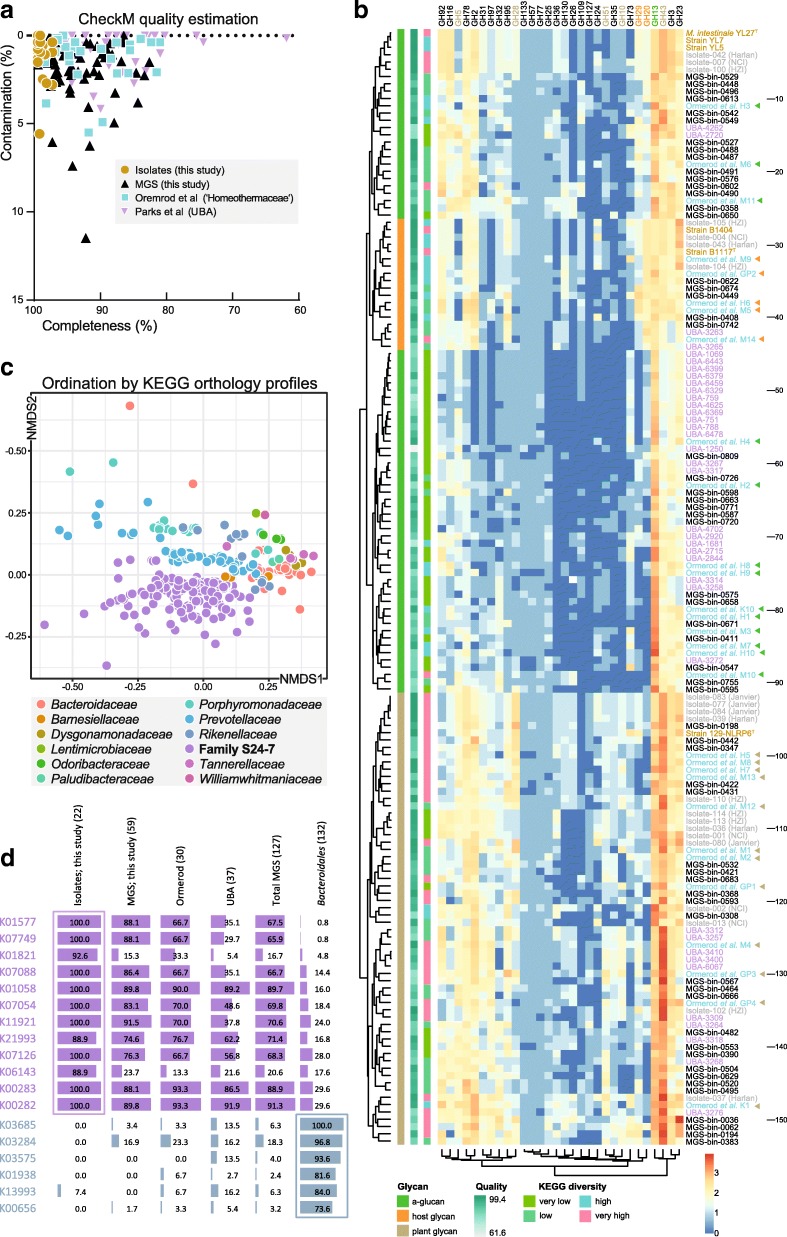
Functional features of family S24-7. **a** Quality plot of the genome sequences used for analysis. Assemblies generated in the present study (isolates and metagenomic species) were considered if the marker gene completeness minus contamination was ≥ 80% [45]. Other reconstructed genomes are from two studies previously published [44, 46]. **b** Non-supervised clustering based on glycoside hydrolases (GH) occurrences across all 153 genomes available (numbered in increments of 10 on the right-hand side) as performed previously [44]. Already published entries are labeled in blue (“Homeothermaceae,” *n* = 30) or violet (UBA, *n* = 37) letters. Those from the present study are in black (MGS, *n* = 59), gray (short-term isolates, *n* = 21), or gold (cultured strains; *n* = 5 novel and 1 type species previously published). For the isolates in gray, names in brackets indicate their facility/vendor of origin (HZI, Helmholtz Center for Infection Research, Braunschweig, Germany; NCI, National Cancer Institute, Maryland, USA; Harlan; Janvier). GH categories (labels on top) considered discriminative between the different functional guilds (a-glucan, host or plant glycans) are colored accordingly (green, orange, and brown, respectively). **c** Multidimensional plotting of family S24-7 members and those from neighboring families based on KEGG orthology (KO). **d** Family-specific functions. The plots depict the prevalence (%) of single KOs across the different genome categories (top labels with numbers in brackets). The twelve KOs in violet (top) are specific for the S24-7 family, the seven bluish KOs (bottom) for the members of other families (see panel **c**) within the order *Bacteroidales*. KO definition are as follows (from top to bottom): K01577, oxalyl-CoA decarboxylase [EC:4.1.1.8]; K07749, formyl-CoA transferase [EC:2.8.3.16]; K01821, 4-oxalocrotonate tautomerase [EC:5.3.2.6]; K07088, uncharacterized protein; K01058, phospholipase A1/A2 [EC:3.1.1.32 3.1.1.4]; K07054, uncharacterized protein; K11921, family transcriptional regulator, cyn operon transcriptional activator; K21993, formate transporter fdhC; K07126, uncharacterized protein; K06143, inner membrane protein creD; K00283, glycine dehydrogenase subunit 2 [EC:1.4.4.2]; K00282, glycine dehydrogenase subunit 1 [EC:1.4.4.2]; K03685, ribonuclease III [EC:3.1.26.3]; K03284, magnesium transporter; K03575, A/G-specific adenine glycosylase [EC:3.2.2.31]; K01938, formate-tetrahydrofolate ligase [EC:6.3.4.3]; K13993, HSP20 family protein; K00656, formate C-acetyltransferase [EC:2.3.1.54]

Aiming towards a comprehensive functional description of S24-7 family members, genomes generated in the present work and others currently available from previously published studies were analyzed. Ormerod et al. were the first to explore the functional diversity of family S24-7 in a broad manner, gathering 30 genomes via MGS assembly [44]. Thirty-seven additional MGS previously classified as *Porphyromonadaceae*, yet belonging to family S24-7, were retrieved from a more recent initiative recovering hundreds of metagenome-assembled genomes of “Uncultivated Bacteria and Archaea” (UBA) [46]. In addition, we generated draft genomes from three strains of two new species and two additional strains of the type species *M. intestinale* (see section “Novel cultured diversity within family S24-7” below) [31]. Additional genomes from 21 strains that could be isolated but failed being maintained in culture (referred to as short-term isolates hereon) were also generated. Moreover, we reconstructed MGS by binning shotgun sequencing reads from mouse gut samples (see the “Materials and methods” section), which provided a collection of 59 reconstructed genomes with a completeness minus contamination of at least 80%. These efforts resulted in a total of 153 draft genomes, the quality of which is displayed in Fig. 2a.

We first aimed at expanding the view of glycoside hydrolase (GH) profiles using this comprehensive array of genomic information presented above. Cluster analysis across 30 GH categories revealed four main clusters (Fig. 2b) driven by the guild classification of S24-7 species according to the degradation of *alpha*-glucan (green; GH13), host glycan (orange; GH20 and 29), and plant glycan (beige; GH5, 10, 28, 43, and 51) as proposed by Ormerod and colleagues [44]. Of note, the a-glucan guild, primarily characterized by a higher occurrence of GH13-related genes, was separated into two distinct groups: 26 genomes, including that of the type species *M. intestinale* and three of the previously studied MGS [44], showed a higher prevalence of genes within the GH92, GH16, GH78, and GH2 families, which encode a variety of mannosidases and rhamnosidases as in members of the host glycan guild, possibly indicating a higher functional versatility of these 26 species (Fig. 2b). To determine whether the predicted utilization of complex polysaccharides was associated with genome-wide functional diversification, we annotated all genomes using the KEGG database (Additional file 1: Table S1). Unsupervised clustering resulted in the detection of four distinct genome groups based on differential completeness of KEGG modules (Additional file 2: Figure S1a). According to ordination analysis, S24-7 members of the a-glucan guild tended to be characterized by genomes of low functional potential (module completeness), which was opposite to the plant glycan guild whilst host glycan utilizers interspaced between these two groups (Additional file 2: Figure S1b).

Next, we aimed to determine the functional specificities of S24-7 family members. Multidimensional analysis of KEGG Orthology (KO) profiles indicated that S24-7-derived genomes formed a cluster well-separated from that of numerous species belonging to neighboring families within the *Bacteroidales*, reflecting specific functional features shared by S24-7 species (Fig. 2c). To identify major functions driving this difference, we searched for single KOs with increased or decreased prevalence between the two groups of bacteria via statistical comparison based on the chi-squared test for independence using the Chi2_contingency command within the Scipy python module. This resulted in the detection of 179 KOs with increased and 153 KOs with decreased prevalence in S24-7 members vs. 132 other *Bacteroidales* (Additional file 3: Table S2), including 18 KOs with > 70% difference in prevalence (Fig. 2d). Two major themes were clearly specific to S24-7 genomes: benzoate resistance and nitrogen utilization. The *praC* gene (K01821), involved in benzoate degradation, was present in 91.3% of the genomes from isolates (vs. 4.5% in other *Bacteroidales*). A similar pattern was observed with both the *alpha* and *beta* subunits of glycine dehydrolase (K00282 and K00283, respectively), previously identified as genes responsive to benzoate-induced acidification in *Bacillus subtilis* [27]. With respect to nitrogen utilization, cyanate may be utilized as a nitrogen source by S24-7 members, as the *cynR* gene (K11921), which regulates transcription of the cyanate utilization operon [5], was identified in all isolates. KOs that were deficient within family S24-7 included a heat-shock protein (K13993) of the HSP20 family acting as chaperone to protect heat-sensitive proteins [58], which may be a contributing factor as to why family members colonize preferentially the intestinal tract of mice and other homoeothermic animals.

Plasmids were reconstructed within 17 of the 27 genomes from isolates (63%), eight of which contained multiple plasmids (up to three). In total, 27 plasmids were identified and contained 409 genes, of which only 85 (20.8%) could be annotated using BlastKOALA. Of those annotated, Brite reconstruction identified 42 as metabolic enzymes and 32 as involved in genetic information processing. The metabolic enzymes performed a diverse range of functions that may provide a survival advantage such as iron storage via ferritin (K02217) and bacterial defense systems (K19158). In order to investigate redundancy of the functional potential encoded on S24-7-derived plasmids, protein clustering was conducted using BLASTP requiring 90% query coverage and 90% identity match. In total, 36 clusters were formed, of which the largest seven clusters each contained 11 proteins with no functional annotation from multiple plasmids. The high occurrence of these protein clusters within the S24-7-derived plasmids suggests they may be of some importance for the family; however, further functional characterization of these proteins is required to validate such insight. One cluster with known function contained four proteins, all annotated as ATP-binding cassette transporters (K06147). These proteins occurred within three different plasmids, indicating some level of redundancy of transport systems across S24-7-derived plasmids.

### Occurrence and featured pathways of selected species

**Fig. 3:**
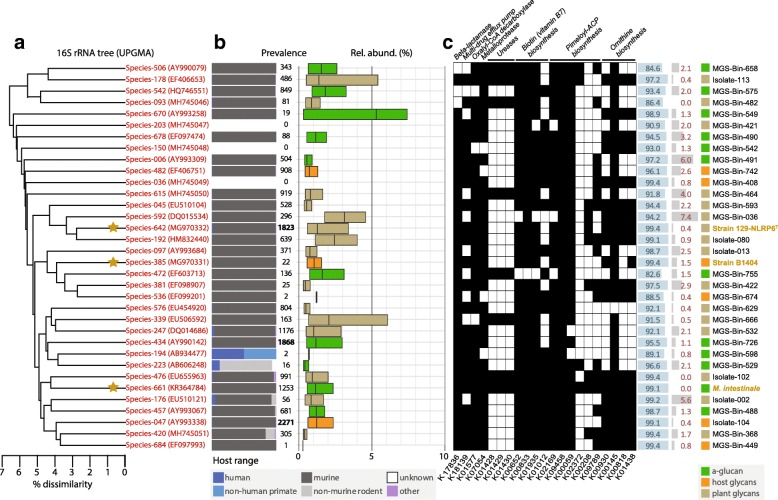
Occurrence, glycan degradation capacities, and specific functional features of selected S24-7 species. **a** UPGMA tree showing the phylogenetic position of the 34 species with both a 16S rRNA gene sequence (used to calculate the tree; see accession numbers) and a draft genome available. Yellow stars indicate cultured species. **b** Occurrence of the species selection as determined by large-scale amplicon analysis using IMNGS (www.imngs.org). Colored bars (gray, blue, violet) indicate the type of samples positive for the given species, the prevalence indicating the number of corresponding samples out of a total of 93,045, including 10,350 from the mouse gut. A sample was considered positive only if sequence similarity matches occurred ≥ 0.25% relative abundance, a threshold of confidence below which the risk of including spurious OTUs in the analysis increases substantially. Relative abundances shown as box plots (median with interquartile range) include data from the positive samples only and are color-coded according to glycan guilds (see panel **c**). **c** Binary presence (black)/absence (white) map of target pathways and single KOs with increased prevalence in S24-7 family members (Fig. 2d and Additional file 3: Table S2). KO and pathway designations are given on the top of the map; KO numbers at the bottom. Blue and red data bar on the right-hand side of the map indicate completeness and contamination values (%) for each of the genomes analyzed

In order to link phylogeny to genomic information, we applied a novel approach to assemble 16S rRNA gene sequences in parallel to MGS reads. In addition, high-quality genomes from three cultured species (six strains in total) could be generated. Thereby, representative genomes of the observed 685 16S rRNA gene-derived species (Fig. 1a) were selected whenever available, resulting in a collection of 34 non-redundant species with draft genomes available (Fig. 3a).

Considering the sample types used as template for metagenomic sequencing and for strain isolation, it was not surprising to observe that the vast majority of these 34 species (85%, *n* = 29) were specific to the mouse gut, including five species (no. 642, 247, 434, 661, and 047; from top to bottom in the tree) represented by more than 1000 positive samples (out of 10,350) and 9 additional species by > 500 samples (no. 542, 006, 482, 615, 045, 192, 576, 476, and 457) (Fig. 3b). One species (no. 194) occurred primarily in human and non-human primates and one other (no. 223) in non-murine rodents; however, these species were represented by low number of samples (*n* = 2 and 16, respectively). Three species (no. 203, 150, and 036) were orphan in terms of ecological niches represented in IMNGS-derived samples, suggesting that they belong to subdominant communities not captured by amplicon sequencing. The maximum relative abundance among any given single mouse-specific S24-7 species across all individual samples was 18.3%, with median values between 0.3 to 5.3% per species (Fig. 3b).

As mentioned in the last section, based on their predicted potential to degrade complex carbohydrates, members of family S24-7 were previously grouped in the three trophic guilds with different degradation capacities: a-glucans, complex plant cell wall glycans (hemicellulose and pectin), and host-derived glycans [44]. However, corresponding 16S rRNA gene-based information was not available and the relative scarcity of mouse metagenomes required for genome-mapping approaches did not allow assessing the occurrence of these guilds comprehensively. Hence, we predicted the carbohydrate utilization profile of the 34 genomes (MGS/isolates) with 16S rRNA gene sequence available and thereby determined the prevalence and relative abundance of members of the three guilds using IMNGS. This prediction identified members of each guild within the 34 selected genomes, with the following prevalence: host glycans (*n* = 6), a-glucans (*n* = 12), and plant glycans (*n* = 16) (Fig. 3c). Interestingly, the species with highest prevalence across all samples (no. 047, 434, and 642; the latter corresponding to strain 129-NLRP6^T^ described taxonomically below) each represented a different guild, highlighting the presence of various carbohydrate-utilization strategies among dominant S24-7 members in the mouse intestine. This agrees with the finding above (Fig. 1e), revealing the co-occurrence of several S24-7 species in a given mouse microbiota. There was no obvious association between the occurrence of species and the functional guild to which they belong, i.e., prevalence distribution and the range of average relative abundances of single species were relatively wide among members each of the guilds.

Statistical analysis of KOs prevalence (Additional file 3: Table S2) identified interesting S24-7-relevant pathways and genes, the occurrence of which within the species selection is depicted in Fig. 3c. Pathways included the biosynthesis of vitamin B7 (biotin) and the amino acid ornithine. All KOs converting pimeloyl-ACP to biotin were identified as enriched within the family, except the final conversion of dethiobiotin to biotin (the KO facilitating this conversion, K01012, was present in 63% of isolates independent of genome quality vs. 43% of other *Bacteroidales*). While only the starting KO of the pimeloyl-ACP biosynthesis module was enriched in S24-7 species, multiple other KOs within this module were present in high numbers, representing therefore the likely route of precursors production for B7 biosynthesis. Future functional studies will be required to characterize the relevance of these pathways in more detail. Ornithine biosynthesis (M00028) contained four enriched KOs which converted *N*-acetyl-glutamate to ornithine. This represents a family-specific pattern of ornithine production that is unique within known *Bacteroidales*. Whereas several previously identified functions [44] such as oxalyl-CoA decarboxylase (K01577) involved in oxalate degradation were confirmed to be widespread across S24-7 species, ureases (K01428-30) were detected in only eight of the 34 species, yet remained specific to the family when compared to other *Bacteroidales* (prevalence < 5%; Additional file 3: Table S2).

### Novel cultured diversity within family S24-7

**Fig. 4:**
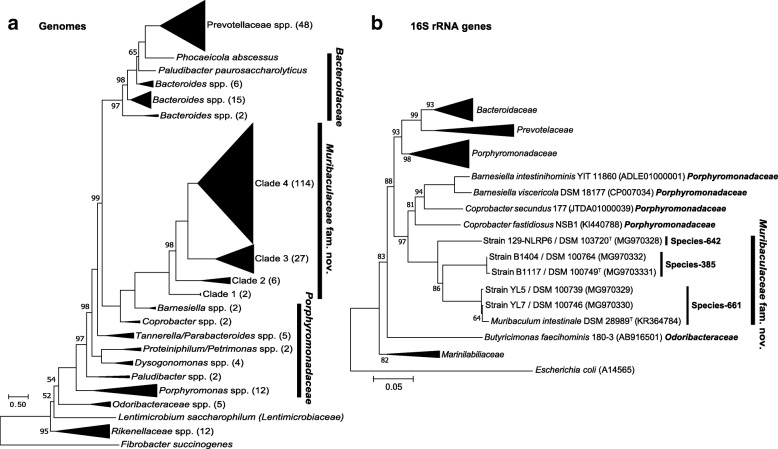
Phylogeny of S24-7 species and novel isolates within the family. **a** Phylogenomic placement of the *Muribaculaceae* family within the phylum *Bacteroidetes* was conducted using PhyloPhlAn [57]. Representative genomes within *Bacteroidetes* were used to place the *Muribaculaceae* isolates and MGS genomes generated in the present study; *Fibrobacter succinogenes* was used as out-group. The tree is drawn to scale with branch lengths measuring the number of amino acid substitutions per site. Local support values were calculated using the Shimodaira-Hasegawa test with 1000 resamples; only those values < 100% are shown. For the sake of clarity, clades with a branch length to nodes < 2 have been collapsed with the size of the triangle being proportional to the number of genomes within the corresponding clade (see Additional file 4: Figure S2 for the original, not collapsed tree structure within *Muribaculaceae*). Clades are named based on the internal genomes taxonomy, with corresponding numbers of genomes indicated in brackets. **b** Tree based on 16S rRNA gene sequences showing the phylogenetic position of cultured species within *Muribaculaceae* compared with members of most closely related families. The evolutionary history was inferred using the Neighbor-Joining method [53]. The optimal tree with the sum of branch length 2.97236459 is shown. The percentage of replicate trees in which the associated taxa clustered together in the bootstrap test (100 replicates) are shown next to the branches (values equal to 100% are not shown) [15]. The tree is drawn to scale, with branch lengths in the same units as those of the evolutionary distances used to infer the phylogenetic tree. The evolutionary distances were computed using the Maximum Composite Likelihood method [64] and are in the units of the number of base substitutions per site. The analysis involved 64 nucleotide sequences. All ambiguous positions were removed for each sequence pair. There were a total of 1518 positions in the final dataset. Evolutionary analyses were conducted in MEGA7 [28]

In light of the wide sequence-inferred diversity aforementioned and to go beyond descriptive molecular work but towards future functional studies on the role of S24-7 family members in gut microbial communities, the final aim of this work was to describe additional cultured taxa within the family. We were able to isolate and maintain in culture a total of five strains from the mouse intestine belonging to family S24-7. Both phylogenomic and 16S rRNA gene sequence analysis of these isolates and related taxa, including all genomes obtained in the present study and those from previous studies [44, 46], clearly showed robust branching of S24-7 members separate from neighboring families within the order *Bacteroidales*, including *Porphyromonadaceae*, *Bacteroidaceae*, *Rikenellaceae*, and *Odoribacteraceae* (Fig. 4). This strongly supports the evolutionary separated standing of family S24-7, for which we propose creation of the name *Muribaculaceae* to accommodate already known species [31], the isolates described below, and all future additional members of family S24-7.

Phylogenomic analysis revealed the presence of four distinct, unevenly distributed clades within *Muribaculaceae* (Fig. 4a), which were not linked to module-based functional profiles (Additional file 2: Figure S1). Clade 4 represented most of the genomes analyzed and included all five isolated strains. According to 16S rRNA gene-based phylogeny (Fig. 4b), strain YL5 (= DSM 100739) and YL7 (= DSM 100746) were additional members of the type species of the type genus (*M. intestinale*; species-661 in Fig. 3b) [31]. Strains B1404 (= DSM 100764) and B1117^T^ (= DSM 100764^T^) formed a distinct cluster (species-385 in Fig. 3b) branching next to *M. intestinale*, whereas strain 129-NLRP6^T^ (= DSM 103720^T^) was the single cultured member of a more distantly related taxon (species-642 in Fig. 3b). Sequence identity values between the 16S rRNA genes of these new taxa and *M. intestinale* were 90.5% (species-385) and 90.1% (species-642). Identity between the two novel taxa was 89.1%. Digital DNA-DNA hybridization (dDDH) values between any of the novel strains among another and with *Barnesiella* and *Coprobacter* spp. were ≤ 31.4%. PCOP values across the same comparisons were ≤ 55.5%. These data support the creation of two novel genera to accommodate the isolated strains, for which the names *Duncaniella muris* (strain 129-NLRP6^T^) and *Paramuribaculum intestinale* (strain B1404 and B1117^T^) are proposed. Taxonomic descriptions are provided below.

## Discussion

As the functions of many mammalian gut bacteria and their interactions with the host are still unknown, there has been a renewed interest in using cultivation techniques for the description of novel diversity [10, 13, 29]. The bacterial family S24-7, previously also referred to as MIB [55] or ‘Homeothermaceae’ [44], is a very interesting target within the order *Bacteroidales* (phylum *Bacteroidetes*) because of its still undescribed status despite widespread occurrence in animal guts [44, 56, 66]. We here provide taxonomic description of this family and studied its sequence-based diversity and ecology.

The relatively high number of studies reporting major shifts in S24-7 family members linked to various dietary treatments, host conditions, or colonization processes in rodents using high-throughput sequencing [8, 43, 56, 59, 60, 67] contrasts to the very few reports on their diversity. The recent work by Philip Hugenholtz and colleagues [44] is the most comprehensive study on S24-7 bacteria available to date. These authors investigated their diversity and functional potential using a set of 30 metagenome-derived genomes representing 27 uncultured species, thereby highlighting their ability to degrade a variety of complex carbohydrates. In comparison, the added value of the present work is threefold: (i) it extends our knowledge of family S24-7 to the analysis of 123 additional draft genomes, thereby revealing novel aspects of S24-7-derived functions; (ii) combines it with large-scale 16S rRNA gene-based assessment of their diversity and ecological distribution; and (iii) provides novel cultured strains that open new avenues for functional studies.

Our integrative 16S rRNA amplicon-based analysis revealed the colonization profiles of S24-7 species at large scales. Findings agree with a previous survey on their occurrence in the intestinal tract of various animals [44, 61], yet with a very clear dominance in the gut of rodents. It is worthwhile noting that, despite their universal aspect, amplicon sequencing databases, and consequently the platform www.imngs.org used for analyses in the present study, are skewed towards certain ecosystems, including human- and mouse-derived body habitats, in particular the gut. Hence, we cannot exclude that studies focusing on other animals will reveal the dominance of S24-7 family members in their gut microbiota, as observed here in pigs and previously in koalas [61]. Nonetheless, the particular adaptation of S24-7 family members to the mouse gut was also shown experimentally by Seedorf and colleagues [56], who found that bacterial taxa of mouse origin, in particular S24-7 species, outcompeted human-derived taxa 14 days after colonization of germfree mice.

Relative abundance values of individual S24-7 species of up to 30% in our study demonstrate their status of dominant gut bacteria, which agrees with literature data referring to up to 15% metagenomic reads for a given species [44]. We also found that at least eight different S24-7 species co-occur on average in the mouse intestine, which speaks in favor of their functional versatility and thus the ability to occupy different niches in common communities. This versatility concerns complex carbohydrates degradation, as shown before [44] and revisited in this study with the suggestion of possibly four GH-based guilds and the release of one cultured species for three of these guilds. This fitness of S24-7 species in degrading dietary carbohydrates most likely explains also the many reports on their decreased occurrence in the context of feeding trials using high-calorie and/or carbohydrate-enriched diets [8, 31, 43]. The ability of different species to use various sources of nitrogen such as urea or cyanate, which both seem to represent a functional particularity within the order *Bacteroidales*, may also account for their specific colonization behavior. Finally, our functional analysis suggested protective mechanisms against benzoate to represent a shared functional feature among S24-7 spp. (> 90% prevalence). Even though benzoate is a widely used preservative and animal food additive occurring also naturally in many fruits and vegetables, widespread exposure of mice to this compound remains to be determined.

The prevalence of S24-7-specific KOs tended to be lower within reconstructed genomes than draft genomes from isolates (Fig. 2d and Additional file 3: Table S2). Hence, despite technical advances over the last years, correct assignment of common genes that occur within multiple genomes, such as resistance genes, can remain problematic during bioinformatic processing of metagenomics reads [1, 16, 42]. This was observed for instance with virginiamycin A acetyltransferase (K18234), which confers resistance to macrolide antibiotics and has a prevalence of 91.3% within the isolates vs. 21.4% in MGS and 22.7% in other *Bacteroidales*. While this suggests that macrolide resistance is common within family S24-7, it highlights the methodological limitations of utilizing MGS alone to study novel taxa and the benefit of maintaining strains in culture and describing them in detail. After providing the first taxonomic description of a cultured S24-7 species in a recently published work [31], we add two novel genera in the present study, one of them representing a prevalent species (nearly 2000 mouse samples positive) of the plant glycan-degrading guild. Despite stringent filtering and clustering parameters used during processing, our sequence-based estimation of 685 species within the family clearly shows the amount of work that remains to be done in order to extend the list of cultured S24-7 species and thereby our understanding of the entire family. At both the functional and phylogenomic level, family S24-7 could clearly be delineated from neighboring families within the *Bacteroidales*. In contrast, an additional effort beyond scope of the present work will be required to clarify incongruent taxonomy within the *Porphyromonadaceae* and validate taxa to accommodate *Coprobacter* and *Barnesiella* spp. [44, 54, 62].

In conclusion, we provide novel insights into the sequence-based and cultured diversity and ecology of the still relatively cryptic bacterial family S24-7. Among the hundreds of estimated species, three were cultured and we propose the name *Muribaculaceae*, after the first described genus *Muribaculum*, to accommodate these and all other species of the family to be cultured in the future. We only started to decipher the functional particularities of *Muribaculaceae* and additional in-depth genetic analysis and functional studies in vivo will be required to understand their precise roles in the specific host-derived ecosystems they colonize.

### Description of *Muribaculaceae* fam. nov.

*Muribaculaceae* (Mu.ri.ba.cu.la.ce’ae. N.L. neut. n. *Muribaculum* a bacterial genus; -*aceae* ending to denote a family; N.L. fem. pl. n. *Muribaculaceae* the *Muribaculum* family)

*Muribaculaceae* previously referred to as MIB (mouse intestinal bacteria) [55] or *Homeothermaceae* [44] in the literature, classified so far as either *Porphyromonadaceae* in RDP [68] or S24-7 in SILVA [50]. Cells are Gram-negative, non-motile, mesophilic, and do not sporulate. Nearest phylogenetic neighbors according to both genome- and 16S rRNA gene-based analysis are *Barnesiella* and *Coprobacter* spp. within the family *Porphyromonadaceae*, which share < 86.5% 16S rRNA gene identity. The maximum POCP value between the genome of any cultured *Muribaculaceae* and *Barnesiella* or *Coprobacter* spp. is 46.4%. dDDH values across the same comparisons range between 20.7 and 39.4%. The family is characterized by G + C contents of genomic DNA between 49.8 and 53.1 mol%, which differs substantially from *Coprobacter* spp. (38 mol%) and *Barnesiella intestinihominis* (44 mol%), but not *Barnesiella viscericola* (52 mol%). Major cellular fatty acids are saturated and mainly include anteiso-C_15:0_. Altogether, these data indicate the separate status of *Muribaculaceae*, represented by the type genus *Muribaculum*.

### Description of *Duncaniella* gen. nov.

*Duncaniella* (Dun.ca.ni.el’la. N. L. fem. dim. n. *Duncaniella* genus named in honor of Dr. Sylvia Duncan for her outstanding contribution to research on gut bacteria)

*Duncaniella* possess all features of the family. The least distant species based on pairwise 16S rRNA gene sequence identity is *M. intestinale* (90.1%). POCP value between the genomes of strain 129-NLRP6^T^ and *M. intestinale* is 53.6%. The dDDH value between both genomes is 31.4%. Major cellular fatty acids are C_14:0_ (ca. 15%), iso-C_15:0_ (ca. 24%), and anteiso-C_15:0_ (ca. 35%). The type species *Duncaniella muris* is one of the most dominant and prevalent S24-7 species studied so far in the mouse gut (up to 6.6% relative abundance and 18% positive samples of 10,350 in total).

### Description of *Duncaniella muris* sp. nov.

*Duncaniella muris* (mu’ris. L. gen. n. *muris* of the mouse)

The species possesses all features of the genus. Reconstruction of metabolic KEGG pathways (35.1% of predicted ORFs annotated) identified the presence of following broad metabolic groupings: carbohydrate metabolism (12), energy metabolism (5), lipid metabolism (2), nucleotide metabolism (2), amino acid metabolism (9), glycan biosynthesis and metabolism (3), metabolism of cofactors and vitamins (9), metabolism of terpenoids and polyketides (1), and biosynthesis of other secondary metabolites (2).

Enzymes for the utilization of lactose (EC:3.2.1.23), sucrose (EC:3.2.1.20), melibiose (EC:3.2.1.22), fructose (EC:2.7.1.4), dextrin (EC:3.2.1.3), and amylose (EC:2.4.1.18) were detected. However, proteins involved in the utilization of xylan and cellulose as previously described in *M. intestinale* were not present. Degradation of starch (EC:2.4.1.1) can subsequently be followed by biosynthesis of Lauroyl-KD02-lipid IV(A), completing the part of lipopolysaccharide biosynthesis (EC: 2.4.1.1, 5.4.2.2, 5.3.1.9, 2.2.1.1, 5.1.3.1, 5.3.1.13, 2.5.1.55, 3.1.3.45, 2.7.7.38, 2.4.99.12, 2.4.99.13, 2.3.1.241). Folate biosynthesis from RNA occurs via initial conversion into guanosine 5′-triphosphate (EC:2.7.7.6) which is then converted into dihydrofolic acid (EC:3.5.4.16, 3.1.3.1, 4.1.2.25, 2.7.6.3, 2.5.1.15, 6.3.2.17/ 6.3.2.12). Dihydrofolic acid can then be directly converted into folate or indirectly via the intermediary production of tetrahydrofolic acid via EC:1.5.1.3.

The production of DAP-type peptidoglycan is suggested by the presence of penicillin-binding protein 1A, penicillin-binding protein 2, and penicillin-binding protein 5/6 which act to cross-link the peptidoglycan molecules. A single plasmid with a length of 59,194 bp was reconstructed. It contained 80 ORFs, only three of which could be annotated against the KEGG database. N-acetylmuramoyl-L-alanine amidase (EC:3.5.1.28, K01448), which cleaves specific cell wall glycopeptides, was identified along with the RecT recombination protein (K07455) and the parB chromosome-partitioning protein (K03497).

Cellular fatty acids are anteiso-C_15:0_ (34.4%), iso-C_15:0_ (23.7%), C_14:0_ (15.1%), anteiso-C_13:0_ (3.6%), 3OH-C_16:0_ (3.4%), iso-C_13:0_ (3.1%), C_16:0_ (2.9%), C_18:0_ (2.7%), iso-C_14:0_ (2.4%), iso-3OH-C_15:0_ (ca. 2.0%), and traces (< 2.0%) of C_9:0_, C_10:0_, C_12:0_, C_13:1_, w9c-C_18:1_. The type strain is 129-NLRP6^T^ (= DSM 103720^T^). Its G + C content of genomic DNA is 50.8 mol%.

### Description of *Paramuribaculum* gen. nov.

*Paramuribaculum* (Pa.ra.mu.ri.ba’cu.lum. Gr. prep. *para*, beside; N.L. neut. n. *Muribaculum* a bacterial genus; N.L. neut. n. *Paramuribaculum* designates relationship to the genus *Muribaculum*)

*Paramuribaculum* possess all features of the family. The least distant species with a valid name based on pairwise 16S rRNA gene sequence identity is *M. intestinale* (90.5%). The POCP value between the genomes of strain B1117^T^ and *M. intestinale* is 53.3%. The dDDH value between both genomes is 28.4% and the difference in G + C mol% is approximately 3%. Relatedness values with strain 129-NLRP6^T^ (*Duncaniella muris*) are < 89% (16S rRNA gene identity), < 55.5% (PCOP), < 26% (dDDH), and > 2% (G + C mol% difference). Altogether, these values clearly indicate a separate genus status. Major cellular fatty acids are anteiso-C_15:0_ (ca. 59%) and w9c-C_18:1_ (10–12%). The type species is *Paramuribaculum intestinale*.

### Description of Paramuribaculum intestinale sp. nov.

*Paramuribaculum intestinale* (in.tes.ti.na’le. N. L. neut. adj. *intestinale* pertaining to the intestine)

The species possesses all features of the genus. Reconstruction of metabolic KEGG pathways (39.6% of predicted ORFs annotated in strain B1117^T^, 41.7% in strain B1404) identified the presence of following broad metabolic groupings: carbohydrate metabolism (12), energy metabolism (6 in strain B1117^T^ and 5 in strain B1404), lipid metabolism (2), nucleotide metabolism (2), amino acid metabolism (9), glycan biosynthesis and metabolism (3), metabolism of cofactors and vitamins (9), metabolism of terpenoids and polyketides (1), and biosynthesis of other secondary metabolites (2).

Enzymes for the utilization of lactose (EC:3.2.1.23), fructose (EC:2.7.1.4), and amylose (EC:2.4.1.18) were detected. However, proteins involved in the utilization of xylan and cellulose as previously described in *M. intestinale* were not present, despite detection of the enzyme responsible for the conversion of cellodextrin to glucose (EC:3.2.1.21). Degradation of chitin (EC:3.2.1.14) may lead directly to *N*-acetyl-D-glucosamine (GlcNAc), or with chitobiose (EC:3.2.1.52) as an intermediate. GlcNAc can be further degraded into fructose-6-phosphate (EC:3.5.1.25, 3.5.99.6). The complete conversion of pyruvate into L-valine (EC:2.2.1.6, 1.1.1.86, 4.2.1.9, 2.6.1.42) was identified uniquely in *Paramuribaculum intestinale* when compared with relatives.

Strain comparison (B1404 vs. B1117^T^) identified 98% of shared annotated KEGG functions. Functions specific to strain B1117^T^ include sulfate adenylyltransferase subunit 2 (EC:2.7.7.4), nicotinamide phosphoribosyltransferase (EC:2.4.2.12), and ornithine cyclodeaminase (EC:4.3.1.12). Functions specific to strain B1404 include histidinol dehydrogenase (EC:1.1.1.23), NADH-quinone oxidoreductase subunit A (EC:1.6.5.3), arginine decarboxylase (EC:4.1.1.19), and alcohol dehydrogenase (EC:1.1.1.-). No plasmid was detected in either of the strains.

The type strain is B1117^T^ (= DSM 100749^T^). Its G + C content of genomic DNA is 53.1 mol%. Cellular fatty acids are anteiso-C_15:0_ (58.7%), w9c-C_18:1_(12.0%), C_16:0_ (10.0%), iso-C_13:0_ (7.2%), C_18:0_ (5.2%), C_14:0_ (1.6%), iso-C_14:0_ (1.3%), and unknown fatty acids (4.0%). B1404 (= DSM 100764) is another strain within the species.

## Additional files

Additional file 1: **Table S1.** Table of all KEGG Orthologies across S24-7 family members and other *Bacteroidales* spp. (XLSX 3531 kb)

Additional file 2: **Figure S1.** KEGG-based analysis of S24-7 functions. A heatmap of differential completeness of KEGG modules across all 153 genomes analyzed in this study (colored as in Fig. 2 according to their type and study of origin). b Ordination analysis based on CAZy profiles showing that the a-glucan and plant glycan guild tended to be associated with genomes of low and high diversity, respectively, independent of genome quality and as assessed via completeness of KEGG modules (numbers in brackets refer to clusters in the heatmap). Multivariate analysis was calculated using the ADONIS function within the R vegan package (Dixon 2003). (PDF 1472 kb)

Additional file 3: **Table S2.** Discriminative KEGG Orthologies between S24-7 family members and other *Bacteroidales* spp. (XLSX 33 kb)

Additional file 4: **Figure S2.** Extended phylogenomic tree as described in Fig. 4 and the corresponding methods. Accessions of the genomes from short-term isolates (gray) are given in brackets next to their mouse facility of origin. (PDF 215 kb)
