# Supplementary figures and images for: Sequence and cultivation study of Muribaculaceae reveals novel species, host preference, and functional potential of this yet undescribed family
Source: Microbiome. 2019 Feb 19;7:28. doi: 10.1186/s40168-019-0637-2 (PMC6381624; doi:10.1186/s40168-019-0637-2)

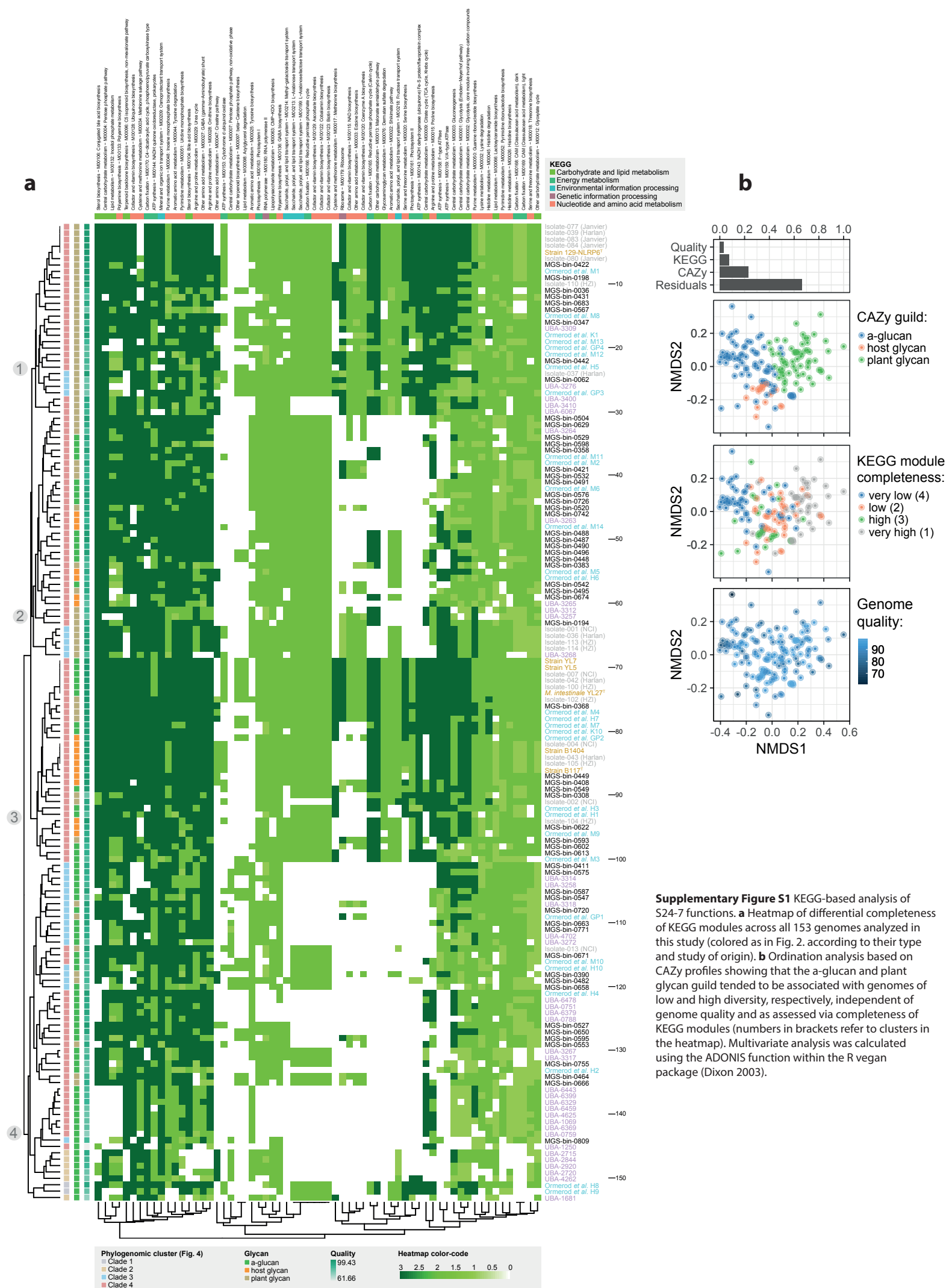

Supplement: Supplementary file 2 — Figure S1. KEGG-based analysis of S24-7 functions. A heatmap of differential completeness of KEGG modules across all 153 genomes analyzed in this study (colored as in Fig. 2 according to their type and study of origin). b Ordination analysis based on CAZy profiles showing that the a-glucan and plant glycan guild tended to be associated with genomes of low and high diversity, respectively, independent of genome quality and as assessed via completeness of KEGG modules (numbers in brackets refer to clusters in the heatmap). Multivariate analysis was calculated using the ADONIS function within the R vegan package (Dixon 2003). (PDF 1472 kb) [file 40168_2019_637_MOESM2_ESM.pdf]

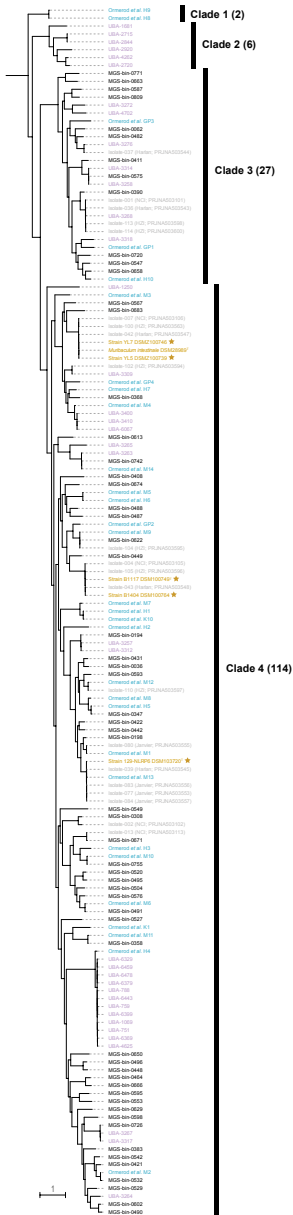

Supplement: Supplementary file 4 — Figure S2. Extended phylogenomic tree as described in Fig. 4 and the corresponding methods. Accessions of the genomes from short-term isolates (gray) are given in brackets next to their mouse facility of origin. (PDF 215 kb) [file 40168_2019_637_MOESM4_ESM.pdf]
